# STA-MCA Bypass as a “Bridge” to Pituitary Surgery in a Patient with an Adenoma Occluding the Internal Carotid Artery: Case Report and Review of the Literature

**DOI:** 10.1155/2015/359586

**Published:** 2015-09-06

**Authors:** Luigi A. Lanterna, Carlo Brembilla, Antonio Signorelli, Paolo Gritti, Emanuele Costi, Gianluigi Dorelli, Claudio Bernucci

**Affiliations:** ^1^Department of Neuroscience and Surgery of the Nervous System, Papa Giovanni XXIII Hospital, Piazza OMS, Organizzazione Mondiale della Sanità 1, 24127 Bergamo, Italy; ^2^Department of Neuroanesthesiology, Papa Giovanni XXIII Hospital, Piazza OMS, Organizzazione Mondiale della Sanità 1, 24127 Bergamo, Italy

## Abstract

Occlusion of the intracranial internal carotid artery (ICA) by a pituitary adenoma with resulting cerebral ischemia is a very rare but devastating occurrence. The authors present a case in which a condition of symptomatic ICA occlusion due to a giant pituitary adenoma was successfully treated using a preliminary extraintracranial bypass as a “bridge” to the tumor removal. A 52-year-old patient presented with a minor stroke followed by pressure-dependent transient ischemic attacks consistent with a condition of hypoperfusion. MR imaging and a digital subtraction angiography revealed a pituitary adenoma occluding the ICA on the right side. He underwent a superficial temporal artery to middle cerebral artery (STA-MCA) bypass with the aim of revascularizing the ischemic hemisphere and reducing the risk of perioperative stroke or stroke evolution. The patient was subsequently operated on to remove the adenoma through a transsphenoidal approach. The postoperative course was uneventful and the patient has suffered no further ischemic events. When there are no emergency indications to decompress the optical pathways but the patient is at risk of impending stroke because of ICA occlusion, a two-step strategy consisting of a bypass and subsequent removal of the pituitary adenoma may be a valuable option.

## 1. Introduction

The association of internal carotid artery (ICA) occlusion and pituitary tumors is a very rare occurrence that has been described in only a handful of case reports [1, 2]. Typically, the ICA occlusion has been attributed to the extrinsic compression exerted by the tumor on the artery that lies in the confined parasellar space. The clinical course is often stormy with a high risk of stroke evolution and mortality. The management strategy has ranged from a conservative approach to attacking the tumor directly. In no case has the option of treating the ischemic risk first and then tackling the tumor in a safer setting been considered. We report the case of a patient with a pituitary adenoma causing symptomatic ICA occlusion in whom we decided to perform a cerebral bypass as a “bridge” to the removal of the adenoma, and we review the literature.

## 2. Case Report

### 2.1. History, Examination, and Diagnosis

**Figure 1:**
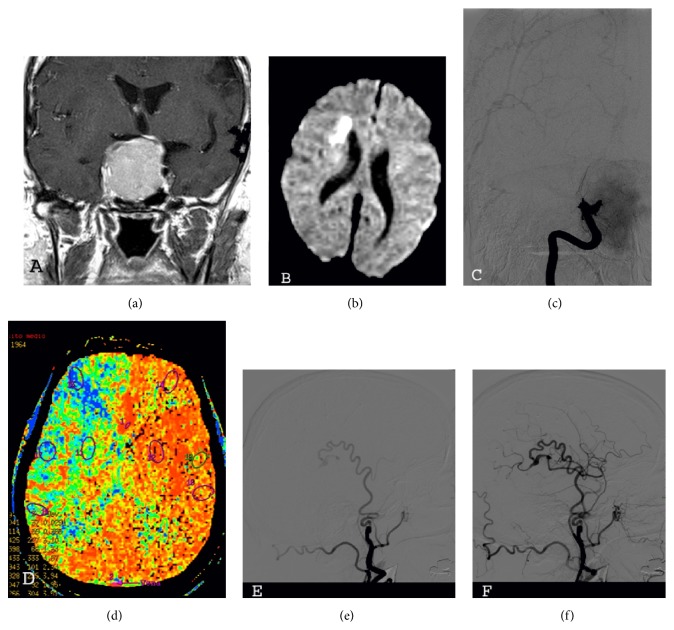
(a) Magnetic resonance showing the pituitary adenoma. (b) Magnetic resonance showing the ischemic lesion on the right side. (c) Digital subtraction angiography (DSA) showing the occlusion of the internal carotid artery at the level of the cavernous sinus on the right side. (d) Perfusion weighted CT scan: prolonged mean transit time on the right hemisphere. ((e) and (f)) Postoperative DSA (lateral view) showing the functioning of the bypass, early and late arterial phase.

A 52-year-old male, while still waiting for the investigations for hypopituitarism to be completed as an outpatient, acutely complained of weakness on the left side. On admission to the emergency department, the neurological examination revealed a hemiparesis on the left side. His visual acuity was intact. He underwent MR of the head that found a small area of restricted diffusion on the right side consistent with acute ischemia, intracranial ICA occlusion on the right side, and a large mass that filled the sella turcica and extended superiorly (Figures 1(a) and 1(b)). There were no signs of apoplexy. As the visual deficits were not acutely deteriorating, we decided to delay the operation and to stabilize the patient. He was adequately hydrated and he gradually recovered from the hemiparesis over the next 3 days. However, despite the initial improvement, he experienced repeated transient ischemic attacks (TIAs) during the next few days. A digital subtraction angiography (DSA) confirmed the complete occlusion of the ICA on the right side with unsatisfactory compensations (Figure 1(c)). A perfusion weighted CT (pCT) scan showed a condition of hypoperfusion as suggested by a prolonged mean transit time (Figure 1(d)) and a pathological response to the Diamox stress test on the right side.

### 2.2. Operation and Postoperative Course

Given the condition of hypoperfusion and the relapsing TIAs suggesting a condition of ongoing hemodynamic instability and a risk of impending stroke, we decided to first perform a superficial temporal artery to middle cerebral artery (STA-MCA) bypass. The patient experienced no further TIAs. DSA demonstrated the functioning of the bypass (Figures 1(e) and 1(f)) and then the patient was operated on to remove the pituitary tumor through a transsphenoidal approach. The postoperative course was uneventful and the bitemporal hemianopsia improved. A scheduled CT angiography performed one year after the procedure showed the patency of the bypass. Subsequently, the patient was followed up yearly as an outpatient for the next 3 years and the Doppler examinations confirmed the function of the bypass.

## 3. Discussion

Mechanical obstruction of the ICA by the expansion of a pituitary adenoma is a very unusual but potentially devastating event where eye-saving and oncological priorities intermingle with a high risk of ischemic stroke and fatality. This case report describes a two-step therapeutic strategy that consists of performing a cerebral bypass in preparation for the removal of the pituitary adenoma causing the symptomatic occlusion of the ICA. This is an alternative to the usual approach of directly removing the pituitary tumor.

A MEDLINE and a manual search of the literature revealed only 14 case reports of pituitary adenoma and ICA occlusion [1–14] (Table 1) and in no case had the authors adopted a policy of performing a bypass as a bridge to stabilize the patient and reduce the risk of perioperative stroke or stroke evolution. According to the literature, 11 out of the 14 patients were symptomatic for cerebral ischemia (78.5%), while the ICA occlusion was an incidental finding in 2 cases. Eleven patients were operated on and the standard policy was to directly attack the adenoma with the aim of removing the tumor and decompressing the ICA during a single stage operation. Although the approach of directly removing the tumor universally opened the carotid artery, the clinical outcome remained poor as 5 out of the 9 symptomatic patients who were operated on either died or remained disabled. Although this might be partly related to the severity of the initial stroke, these results raise concerns about this strategy and suggest that just opening the ICA by removing the tumor may sometimes be hazardous.

Although the risk of perioperative stroke or stroke evolution in an unselected population is generally assumed to be very low and the problem of prevention somewhat trivial, this is not true for those patients who have a complete occlusion of the ICA or a symptomatic cerebrovascular disease as in our patient [15, 16]. This problem has been extensively investigated in cardiac surgery where a complete ICA occlusion not amenable to be treated either by carotid endarterectomy or stenting has been found to increase the risk of perioperative stroke more than 6 times [15, 16]. Indeed, the risk further increases when the ICA occlusion is symptomatic as it is a sign of a more labile hemodynamic reserve [17]. Similar results have been found in the setting of general surgery [15–18].

Furthermore, several factors may specifically increase the risk of perioperative stroke or stroke evolution in patients undergoing surgery for pituitary adenomas. Firstly, pituitary adenoma surgery is usually performed under controlled hypotension that may increase the risk in patients who are in the acute phase of an ischemic stroke and who have an exhausted autoregulatory reserve. Secondly, patients with pituitary adenoma may suffer from diabetes insipidus [19], and volume contraction increases blood viscosity and the risk of stroke. Thirdly, in addition to the general procoagulating changes that are induced by any surgical procedure [20] and the impaired fibrinolysis associated with general anesthesia [21], prolactin independently activates the coagulation cascades and is a potent platelet aggregation coactivator [22].

Although the recent extracranial-intracranial bypass trial has not proven the effectiveness of STA-MCA bypass in stable, chronic patients with ICA occlusion, several studies have found that the bypass may improve the autoregulatory reserve in patients with hemodynamic insufficiency [23]. Indeed, the stroke risk profile of our patient who had recently complained of ischemic symptoms and was on the point of undergoing a hypotensive and metabolic challenge (e.g., tumor removal) is thought to be higher and incomparable to that of the patients included in the trial. In addition, recent studies including patients in the acute phase, when the risk of short-term stroke evolution or recurrence is higher, have found a clinical benefit from the procedure [24]. This is consistent with our experience in patients with moyamoya disease (34 hemispheres treated), where 5 out of the 12 patients with fixed deficits improved, the risk of temporary deficits was 5.8%, and no patient developed definitive deficits or died because of the procedure (Lanterna L. A.: moyamoya disease and moyamoya-like; diagnostic work-up and surgical approach. Preliminary experience at the Papa Giovanni XXIII Hospital of Bergamo. Paper presented at the 7th European Japanese Stroke Surgery Congress, Verona, Italy, June 2014).

In conclusion, patients with pituitary adenoma and ICA occlusion represent a clinically heterogeneous population. When the main complaint of the patient is related to cerebral hypoperfusion and visual pathway decompression is not an emergent priority, a STA-MCA bypass as a bridge to tumor removal may be a valuable option.

## Figures and Tables

**Table 1:** Literature review^*∗*^.

Author (year)	ICA-related symptoms	Treatment	Outcome
Schnitker and Lehnert (1952) [3]	Stroke	Conservative care	Death
Sakalas et al. (1973) [4]	No ischemic symptoms	Open surgery in the acute phase	Good recovery
Rosenbaum et al. (1977) [5]	Stroke	Open surgery in the acute phase	Death
Bernstein et al. (1984) [6]	Confusion, hemiparesis	Transsphenoidal surgery in the acute phase	Good recovery
Clark et al. (1987) [7]	Stroke	Conservative care	Severe disability
Lath and Rajshekhar (2001) [8]	Stroke	Transsphenoidal surgery in the acute phase	Death
Yang et al. (2008) [2]	Stroke	Transsphenoidal surgery in the acute phase (4 days)	Good recovery
Chokyu et al. (2011) [1]	Stroke	Delayed transsphenoidal surgery	Disability (hemiplegia)
Schnur and Clar (1989) [9]	Stroke	Open surgery in the acute phase	Good recovery
Cavalcanti and Castro (1997) [10]	Stroke	Open surgery in the acute phase	Good recovery
Yaghmai et al. (1996) [11]	No ischemic symptoms	Transsphenoidal surgery in the acute phase	Good recovery
El-Zammar and Akagami (2010) [12]	Stroke	Open surgery in the acute phase	Severe disability
Spallone (1981) [13]	Stroke	Conservative care	Severe disability
Dogan et al. (2008) [14]	Stroke	Open surgery in the acute phase	Death
Present case	Stroke, TIAs	STA-MCA bypass and delayed transsphenoidal surgery	Good recovery

^*∗*^TIA: transient ischemic attack; STA-MCA: superficial temporal artery to middle cerebral artery bypass.
